# Integrative Approaches to Atopic Dermatitis: Exploring Biomarkers, Current Therapies, and the Potential Role of Osteopathic Manipulative Medicine

**DOI:** 10.3390/ijms27177973

**Published:** 2026-09-07

**Authors:** Stephanie Sawicki, Anjali Dhawan, Dikshya Devkota, Michelle Elway, Anish R. Maskey

**Affiliations:** 1 Department of Microbiology & Immunology, Touro College of Osteopathic Medicine, Great Falls, MT 59404, USA; ssawicki@student.touro.edu (S.S.); adhawan@student.touro.edu (A.D.); 2 Department of Pediatrics, Oklahoma Children’s Hospital OU Health, Oklahoma City, OK 73104, USA; dikshya-devkota@ou.edu; 3 Department of Primary Care & Osteopathic Medicine, Touro College of Osteopathic Medicine, Great Falls, MT 59404, USA; melway@touro.edu; 4 Department of Pathology, Microbiology & Immunology, New York Medical College, Valhalla, NY 10595, USA

**Keywords:** atopic dermatitis, eczema, biomarkers, current therapies, integrative medicine, osteopathic manipulative medicine, skin barrier dysfunction, pruritus

## Abstract

Atopic dermatitis (AD) is a chronic, relapsing, and remitting condition that affects approximately 10% of the population. It is a multifactorial disease involving immune dysregulation, gene mutations, and environmental factors that ultimately disrupt skin barrier restoration, causing pruritic lesions. Given the marked heterogeneity of AD and the presence of multiple immune and clinical endotypes, no single therapeutic strategy is likely to be universally effective. Instead, a growing panel of distinct biomarkers offers an avenue for true precision medicine by enabling more accurate endotype classification, prediction of treatment response, and tailored treatments. In this context, ongoing research is increasingly focused on individualized, biomarker-informed strategies, as well as the evaluation of adjunctive approaches that can be integrated with standard therapies to optimize long-term disease control. Given the central role of systemic inflammation and barrier dysfunction in AD, the mechanistic focus of osteopathic manipulative medicine (OMM) on circulation, lymphatic drainage, and autonomic regulation offers another biological treatment to use alongside conventional therapies. This concise review examines the pathogenesis of AD, current treatments and their limitations, and the expanding role of biomarkers in refining diagnosis, prognosis, and therapeutic decision-making. It also explores the potential integration of OMM as a biologically plausible adjunct to support individualized, endotype-aware treatment strategies, while emphasizing the current lack of empirical evidence and the need for rigorous mechanistic and clinical studies.

## 1. Introduction

Atopic dermatitis (AD), the most common form of eczema, is a chronic inflammatory skin condition affecting an estimated 12.7% of children and 7.6% of adults in the United States, corresponding to approximately 9.2 million children and 19.8 million adults; pediatric prevalence has risen significantly since 2021 [1]. It most commonly begins in early childhood, with up to 60% of cases presenting within the first year of life and approximately 85% before five years of age, coinciding with a critical period of skin barrier and immune system development [2]. It is characterized as persistent immune-mediated inflammation, requiring multiple systemic treatments that vary among individuals. Associated symptoms include erythema, edema, pruritis, xerosis, and scaly eruptions with vesicles, crust formation and erythematous skin, most commonly affecting the flexural surfaces of the body [3]. Other signs include lichenification and, in severe cases, oozing and crusting of the affected areas [4]. These symptoms range from moderate to severe and vary from patient to patient with some experiencing chronic itching and sleep disturbances [5]. The pathogenesis of atopic dermatitis (AD) is multifactorial, arising from interactions among epidermal barrier disruption, immune dysregulation, alterations in the skin microbiome, and environmental exposures—including pollutants, microbial contact, and climatic triggers such as low humidity and abrupt weather change. Genetic contributions to barrier failure are best characterized by filaggrin (FLG), whose loss-of-function mutations are associated with a compromised epithelial barrier and elevated immunoglobulin E (IgE). A second barrier gene, SPINK5, encodes the serine protease inhibitor LEKTI, which restrains the epidermal kallikrein cascade governing desquamation; complete loss of function causes Netherton syndrome—a monogenic disorder of severe barrier failure in which atopy is a near-universal accompaniment—and common SPINK5 coding variants have been associated with AD susceptibility [6,7,8]. AD frequently co-occurs with other atopic conditions, ranges in severity from mild to severe, and typically requires lifelong symptom management [9].

Although serum IgE is frequently elevated in atopic dermatitis, it is not routinely used in practice as a diagnostic or severity marker, as levels correlate inconsistently with disease activity and remain normal in the intrinsic (non-atopic) subset of patients, which comprises roughly 20% of cases [10]. Nonetheless, IgE status remains clinically meaningful as a basis for phenotypic classification. AD can be divided into two subtypes based on IgE levels: extrinsic and intrinsic. Extrinsic AD accounts for about 80% of cases, while intrinsic AD represents the remaining 20%. Although both subtypes exhibit Th2-driven inflammation, intrinsic AD is more strongly associated with Th1 and Th17 activation, with increased IFN-γ-producing cells in the blood [9]. Extrinsic AD, in contrast, is characterized by a disrupted skin barrier, increased transepidermal water loss (TEWL), and filaggrin (FLG) gene mutations, all of which confer a higher risk of eczema and food allergies. Most patients with AD exhibit a Th2-dominant immune response, characterized by secretion of the cytokines IL-4, IL-13, and IL-31 [9]. These cytokines impair skin barrier function by downregulating FLG expression and increasing IgE production, thereby promoting allergic sensitization. Th2 endotypes are associated with increased eosinophilia and pruritus, with elevated IL-13 production contributing to skin barrier dysfunction and inflammation [9]. To counter this, therapeutic agents such as dupilumab target this pathway by preventing IL-13 from binding to its receptor. In more chronic stages of AD, non-Th2 cytokines such as IL-17, IL-22, and TNF-α become increasingly important [11]. IL-22 promotes reversible epidermal hyperplasia and alters the skin microbiota by increasing keratinocyte proliferation, making it an attractive therapeutic target for modulating skin changes in chronic AD. In patients with AD, the skin microbiota is often disturbed by colonization with *Staphylococcus aureus* (*S. aureus*). *S. aureus* can further promote cutaneous inflammation through virulence factors and intracellular signaling pathways that directly affect keratinocytes. α-Toxin forms pores within the plasma membrane of keratinocytes, disrupting the monovalent ion homeostasis, leading to ATP depletion, impaired mitochondrial function, and cellular injury [12]. In addition, PSM-α peptides can induce keratinocyte damage and release alarmins such as IL-1α and IL-36α [13]. These signals through the IL-1R and IL-36R/MyD88 pathways promote IL-17-mediated cutaneous inflammation. Furthermore, *S. aureus* activates TLR2-dependent NF-κB signaling in human keratinocytes, leading to an increased expression of IL-8, COX-2 and iNOS, all of which are inflammatory mediators [14]. Similarly, EGFR signaling also contributes to the keratinocyte response to *S. aureus*. EGFR blockade decreases *S. aureus*-induced IL-1α and IL-1β expression and secretion, as well as inflammasome components such as caspase-1 and ASC, thereby reducing downstream cytokine release [14]. Together, these mechanisms demonstrate how *S. aureus* can interact directly with keratinocytes to increase inflammatory signaling relevant to AD. In pediatric patients, the skin barrier is particularly vulnerable during early development, as key structural proteins such as filaggrin are still maturing, making infants and young children especially susceptible to colonization by *S. aureus* and subsequent barrier disruption [15,16]. *S. aureus* colonization in AD exacerbates thymic stromal lymphopoietin (TSLP) and Th2/Th17-type inflammations [17]. Proposed risk factors for *S. aureus* colonization include increased bacterial adhesion to corneocytes, reduced antimicrobial peptides, decreased filaggrin and its degradation products, elevated Th2 cytokines, microbial dysbiosis, and altered lipid composition.

Another notable feature of AD is the complex sensory experience of pruritus, which is mediated by specialized neural circuits and distinct molecular pathways. While itch serves the biological purpose of alerting the body to irritants or pathogens, when it becomes chronic, it can severely diminish quality of life. Researchers have identified pruritus as a result of complex interactions among itch-specific neurons, immune cells, and inflammatory mediators. IL-31, produced by Th2 lymphocytes, is a key pruritogenic cytokine [18]. It is also expressed in NP3 sensory neurons that express TRPV1 in the dorsal root ganglion, as well as in keratinocytes, innate immune cells, eosinophils, T lymphocytes, and peripheral cutaneous nerve fibers. IL-31 binds its receptor complex and activates JAK1/JAK2, leading to phosphorylation of STAT1, STAT3, and STAT5. Because its receptor is widely expressed, IL-31 can act on the nervous system, immune cells, epithelial surfaces, and stromal cells, contributing broadly to pruritus and inflammation [19].

Due to this heterogeneity and symptom variation from patient to patient, identifying effective treatments remains challenging. Current treatments, including corticosteroids, immunomodulators, biologic injections, topical calcineurin inhibitors, phototherapy, emollients, and antibiotics, primarily function to manage symptoms and often fail to address the underlying causes of the condition specifically in those individuals with high sympathetic skin tone which triggers chronic itch under stress [19,20]. Additionally, a wide array of target biologics are available for AD, however, they are not able to meaningfully treat the symptoms of AD in a substantial subset of patients. Furthermore, biologics are expensive, and relapse is common after withdrawal. This clinical and immunologic heterogeneity has stimulated interest in biomarker-driven, personalized treatment strategies and complementary modalities that address the complex immune pathophysiology of AD [21]. Recent studies show that the use of complementary integrative medicine, including traditional Chinese medicine (TCM) and acupuncture, improves skin lesions, sleep loss, and clinical efficacy of itch [21]. Therefore, using OMM as a whole-body, structure–function-based approach to influence autonomic regulation, lymphatic circulation, and systemic inflammation could be another avenue for the management of chronic stress-activated itch response in AD. This comprehensive review synthesizes current understanding of AD pathogenesis, biomarkers, and targeted therapies and proposes a mechanistically plausible framework in which OMM may serve as an adjunctive strategy to address neuroimmune and lymphatic contributors not fully targeted by cytokine blockade.

## 2. Pathogenesis of Atopic Dermatitis

### 2.1. Barrier Dysfunction

The epidermis serves as the first line of defense against pathogens, allergens and irritants. This protective barrier is composed of lipid-rich lamellar membranes containing ceramides, cholesterol, and free fatty acids [11]. Disruption of any of its molecular components, including proteins and lipids, can compromise the barrier. When this barrier malfunctions, the skin becomes more prone to colonization by pathogenic microbes. Patients with AD experience a noted reduction in microbial diversity, with an overpopulation of strains like *S. aureus* and *Malassezia furfur* (*M. furur*) [11]. These strains trigger an immune response, contributing to the chronic nature of the condition [4]. The human skin microbiota naturally exists in an acidic environment with limited nutrients. Although markedly different from the nutrient-rich environment of the gut, the skin microbiome plays an equally vital role in immune regulation and preventing the colonization of pathogenic organisms. The composition of skin microbiota is affected by factors such as age, anatomical location, and environmental conditions [4]. Individuals with AD experience a significant disruption in the balance of the skin microbiota, often characterized by overgrowths of specific strains of bacteria. *S. aureus* pathogenesis in AD acts by inhibiting the growth of beneficial bacteria such as *S. epidermidis* and Corynebacterium. This dysbiosis contributes to skin barrier dysfunction, heightened inflammatory responses, and an increased risk of recurrent skin infections. This microbial imbalance, driven by *S. aureus*, involves virulent factors such as alpha toxin and gamma toxin, which degrade antimicrobial peptides (AMPs) secreted by commensal bacteria, thereby weakening the skin’s innate defenses. Additionally, the overgrowth of *S. aureus* reduces beneficial bacteria that are crucial for maintaining skin homeostasis. Furthermore, immune responses dominated by Th2 and Th17 pathways further impair AMP production, worsening skin dysbiosis and allowing *S. aureus* to continue colonizing AD lesions [4]. This ongoing cycle leads to the hallmark features of AD, including inflammation and a compromised skin barrier.

The skin barrier in AD is also affected by thymic stromal lymphopoietin (TSLP), an immune regulator that is positively associated with barrier disruption in AD. TSLP is secreted by keratinocytes and subsequently interacts with dendritic cells, T cells, and mast cells, promoting a Th2-mediated immune response in the pathogenesis of AD. TSLP is expressed in the skin, intestine, thymus, lung, and tonsils, and is produced by epithelial and stromal cells, dendritic cells, and mast cells, but is rarely detected in hematopoietic or endothelial cells. Activated skin cells release TSLP, IL-25, and IL-33 from keratinocytes, promoting Th2 responses via type 2 innate lymphoid cells [11]. Following exposure to proinflammatory cytokines (IL-1β and TNF-α) and TLR ligands (TLR2, TLR8, TLR9), TSLP gene expression is upregulated in human airway epithelial cells. Serum TSLP levels in both adults and children with AD are significantly higher than in healthy individuals, and TSLP gene polymorphisms are associated with an increased risk of AD development and progression. As noted, keratinocytes—abundant in the epidermis—are a major source of TSLP and thus play a direct role in promoting AD. Early identification of biomarkers such as elevated TARC/CCL17 and altered stratum corneum lipid composition may allow for earlier intervention, potentially altering disease trajectory before significant barrier and immune dysregulation becomes established [22]. Because TSLP drives pruritus through Th2-mediated pathways, it represents a promising target for therapies aimed at preventing AD progression [23].

### 2.2. Immune System Activation

Antigen-presenting cells in the skin express innate immune receptors known as Toll-like receptors (TLRs). Activation of these TLRs can occur due to tissue damage or the presence of microbes, leading to the stimulation of inflammatory Th2 cells. These Th2 cells then release cytokines like IL-4 and IL-13, which further promote inflammation [24]. While any of these factors alone can contribute to the development of allergic eczema, the disease typically results from a combination of multiple contributing factors. Additionally, IgE autoreactivity has been identified as a key immune pathogenic factor in AD, with IgE autoantigens—primarily intracellular human proteins identified from epithelial cDNA expression libraries—serving as its main targets [25]. In AD, autoantigens like Hom s1-5 and DSF70 are released with elevated IgE levels [24]. These autoantigens trigger mechanical reactions such as scratching, involving mast cells, basophils, T cells, and IgE [26]. This pathway is evidenced by the decrease in IgE autoallergen levels following successful AD treatment. Consequently, immune responses initially triggered by environmental allergens may be sustained by endogenous human antigens.

### 2.3. Microbiome Disruption

As the body’s outermost layer, the skin acts as the first barrier against external pathogens and environmental stressors. At the same time, it is home to a diverse community of bacteria, fungi, viruses, and microeukaryotes—collectively known as the skin microbiome—most of which exist as commensal organisms [27]. The skin offers a supportive habitat for microbial communities, promoting both competition and cooperation while providing essential nutrients. This skin microbiome defense occurs through the production of antimicrobial peptides (AMPs) and other factors that directly inhibit pathogens or modulate their virulence. It has been shown that, in AD, skin microbiota is markedly dysregulated, with altered composition and abundance of surface microbial communities, accompanied by abnormal host immune responses and increased susceptibility to secondary infections [27]. The microbiota, such as *S. aureus* and *S. epidermidis*, directly modulates keratinocyte differentiation and function, thereby shaping adaptive immune responses. Endogenous AMPs, mainly produced by keratinocytes, play a key role by directly inhibiting colonization and infection by pathogenic microorganisms [28]. In addition, a dynamic equilibrium of microbial species and abundance is sustained through both inhibitory and synergistic interactions among cutaneous microbial populations. The skin–microbiome axis is therefore fundamental to maintaining skin homeostasis across the lifespan. However, this relationship is finely balanced, and disturbances can lead to dysbiosis and contribute to the development of skin disorders [27]. *S. aureus* contributes through a self-reinforcing circuit rather than overgrowth alone. α-Toxin is directly cytotoxic to keratinocytes—an effect amplified in cells previously exposed to TH2 cytokines—and induces AD-like inflammation and barrier disruption in proportion to disease severity, while PSMα peptides stimulate proinflammatory cytokine release from keratinocytes [17,29]. This keratinocyte injury drives release of the alarmins IL-33 and TSLP, promoting ILC2 accumulation and amplifying type 2 inflammation [30]. In parallel, the staphylococcal protease V8 cleaves PAR1 on sensory neurons to evoke itch directly, independent of inflammation, and the resulting scratching further degrades the barrier, favoring continued colonization and closing the loop [31].

### 2.4. Lymphatic Imbalance

Lymphatic vessels play a vital role in preserving skin health by limiting inflammation, controlling infection, influencing skin-cancer-related processes, and helping to slow skin aging. Although the underlying mechanisms are complex and not yet fully understood, current evidence shows that an intact cutaneous lymphatic system—modulated by multiple cytokines—helps resolve inflammation, reduces lymphedema, and prevents lymphatic stasis [32]. Obesity is a significant risk factor for atopic dermatitis and psoriasis, in part because these diseases impair lymphatic function—promoting lymphatic leakiness, reduced lymph flow, and lymphatic stasis—which sustains cutaneous inflammation and increases disease severity. Earlier studies have demonstrated that obesity in mice is associated with dysfunctional lymphatics, including increased leakiness of capillary lymphatic vessels and reduced contractile activity of collecting lymphatics [33]. These mice also showed exaggerated dermatitis in response to inflammatory skin challenges, with greater peak inflammation and a slower resolution of the inflammatory response. This hypothesis is supported by prior studies showing that VEGFR-3 blockade and impaired lymphangiogenesis significantly worsens both acute and chronic inflammation, including chronic hypersensitivity reactions induced by DNFB in pulmonary and cutaneous models [33]. These findings suggest that therapies aimed at enhancing lymphatic function could offer a novel approach for treating inflammatory skin disorders.

### 2.5. Sensory Neuron–Immune Crosstalk

Emerging evidence suggests that a disrupted neuroimmune axis, manifested by peripheral neuronal sensitization, epidermal barrier impairment, and altered central itch processing, plays a key role in disease initiation, persistence, and clinical variability [34]. Neuroimmune mechanisms that drive AD involve cytokines such as IL-31, IL-4/IL-13, TSLP, and IL-33, together with neuropeptides including substance P, CGRP, and VIP, which collectively establish self-reinforcing itch–scratch and inflammation loops at the level of both the skin and the central nervous system. Immune-derived mediators, including IL-31 and other type 2 cytokines, can directly activate or sensitize itch-specific neurons, lowering their activation threshold and driving chronic pruritus [34]. These bidirectional neuroimmune interactions sustain an itch–scratch–inflammation cycle that worsens barrier damage and promotes chronic disease. In parallel, psychosocial stress is now recognized as a major exacerbating factor that links the central nervous system to cutaneous immune dysregulation. IL-31 is primarily produced by activated Th2 cells and binds to IL-31RA/OSMR complexes on sensory neurons [35]. This engagement activates the JAK/STAT signaling pathway, enhancing neuronal excitability and upregulating itch-related genes. Clinical studies support this mechanism: in patients with AD, elevated IL-31 levels strongly correlate with itch severity, and pruritic skin shows higher IL-31 expression than non-pruritic areas [35]. Increased IL-31 also promotes the release of brain natriuretic peptide (BNP) from dorsal root ganglion neurons, which stimulates keratinocytes to produce additional cytokines, thereby amplifying cutaneous inflammation [34,35]. By activating the hypothalamic–pituitary–adrenal axis and the sympathetic nervous system, stress alters neuroendocrine mediators such as cortisol and catecholamines, disrupts brain–skin homeostasis, enhances mast cell activation and cytokine release, and thereby intensifies pruritus, inflammation, and flares in AD. This neuroimmunological framework helps explain the strong association between AD severity, sleep disturbance, anxiety, depression, and impaired quality of life. Together, impaired barrier function, abnormal immune activation, microbiome disruption, lymphatic imbalance, and neuroimmune stress responses interact to perpetuate inflammation and pruritus, driving the onset and worsening of AD skin lesions [36].

## 3. Biomarkers in AD

### 3.1. Diagnostic Biomarker

A biomarker is a measurable characteristic that indicates normal biology, disease processes, or responses to an exposure or treatment [37]. Biomarkers can be used to identify individuals at risk (screening), confirm active disease (diagnostic), predict recurrence or progression (prognostic), identify patients most likely to benefit from a therapy (predictive), and monitor treatment effects, disease severity, drug responses, and side effects (pharmacodynamic) [37]. They may derive from genomics, transcriptomics, proteomics (such as cytokines and chemokines), or morphology (such as immunohistochemistry) and can be measured in blood, saliva, urine, or tissue samples, including skin biopsies and tape strips [37]. Biomarkers are increasingly recognized as essential tools for characterizing the heterogeneity of AD, which most experts view as comprising multiple phenotypes and endotypes [38]. This heterogeneity shows differences in epidermal barrier dysfunction, immune activation, genetic susceptibility, and inflammatory signaling in the patient population. As a result, no single biomarker currently captures the full biological complexity of AD, and combinations of molecular markers may provide a more comprehensive characterization of the disease. Surveys from the International Eczema Council show that clinicians already use markers such as IgE and eosinophils, with growing interest in FLG, LDH, and CCL17/TARC, to support diagnosis, monitor compliance, and predict therapeutic response [38]. However, IgE and eosinophilia are not specific to AD and can vary among patients, limiting their use as standalone diagnostic biomarkers [38]. Several biomarkers, including NOS2 and CCL27/CTACK, help distinguish AD from psoriasis, while alterations in stratum corneum lipids, elevated CCL17/TARC, low vascular endothelial growth factor, and high IgE levels have been linked to disease onset or persistence [39].

### 3.2. Severity of Biomarker

Recent studies using predictive models have classified pediatric and adult AD into distinct inflammatory endotypes, with markers such as PARC/CCL18, apelin, TNF-β, MCP-3, IL-13, and IL-5 correlating with severity and chronicity [40]. These findings suggest that patients with similar clinical disease severity may have distinct underlying inflammatory profiles. Molecular biomarkers can complement clinical severity measures such as EASI and SCORAD by providing additional information about the biological activity of disease [40]. Collectively, these findings support the use of biomarkers to refine diagnosis, stratify patients, and guide targeted, personalized therapies in AD. Biomarkers are key tools in advancing precision medicine for AD, informing prevention and guiding the development of new therapies under strict regulatory standards [41]. Markers such as inducible nitric oxide synthase (NOS2) and the chemokine CCL27 (CTACK) reliably distinguish atopic dermatitis from psoriasis, improving diagnostic accuracy and management, especially in psoriasiform dermatitis. Markers of systemic inflammation (e.g., serum lactate dehydrogenase, C-reactive protein) and allergy (e.g., peripheral eosinophil counts) are under investigation in AD [41]. Because AD is largely driven by TH2 and TH22 responses, related cytokines and chemokines have been studied as candidate biomarkers. IL-13 and IL-22 are key contributors to pathogenesis, while chemokines such as CCL17 (TARC), CCL26 (eotaxin-3), CCL27 (CTACK), CCL18, and CCL22 (MDC) correlate with disease severity and may serve as markers of disease activity and progression [41].

### 3.3. Predictive Biomarker

Preventive and prognostic biomarkers play a central role in managing AD, as they are essential for understanding disease trajectory in the context of its complexity and the distinct ways it affects specific patient subgroups. Prognostic and predictive biomarkers provide related but distinct information. Prognostic biomarkers can estimate the likelihood of disease development or persistence whereas predictive biomarkers may identify patients more likely to respond to a specific therapeutic intervention. Therefore, because AD can manifest with varying severity and persist throughout a person’s life, biomarkers are crucial for predicting disease progression and may serve as potential targets for treatment [41]. Prognostic biomarkers for AD development include altered stratum corneum lipid composition and elevated thymus and activation-regulated chemokine (TARC)/chemokine C-C motif ligand 17 (CCL17) levels in both skin tape strips and umbilical cord blood [37]. Recent evidence highlights several biomarkers linked to disease persistence and systemic involvement in AD. Low serum vascular endothelial growth factor levels have been associated with persistent disease in children at 7 years of age, whereas elevated serum IgE levels in adults correlate with ongoing eczema after 10 years [42]. Periostin, an extracellular matrix protein upregulated by the Th2 cytokines IL-4 and IL-13—hallmarks of AD immune responses—has emerged as another key marker [40]. Along with IL-13 and dipeptidyl peptidase-4 (DPP-4), periostin can be used to assess the systemic impact of skin inflammation in AD, and notably, many patients with relatively low Eczema Area and Severity Index (EASI) scores (<16) still exhibit elevated levels of these biomarkers [40]. Identifying such predictive factors and biomarkers when selecting therapy will support more tailored, personalized care and, ultimately, better outcomes for patients with AD. Endotypic profiles in AD vary meaningfully by age and ancestry, adding a further layer of heterogeneity to the biomarker signatures described above. Pediatric disease shows comparatively less TH1 activation, with barrier dysfunction driven substantially by defects in epidermal lipid metabolism, whereas adult disease engages the TH22, TH17, and TH1 axes against a characteristically weakened epidermal barrier [15]. European American patients demonstrate greater differential TH2/TH22 activation with lower TH1/TH17 expression and suppression of filaggrin (FLG) and loricrin, while Asian patients exhibit accentuated TH22/TH17 polarity and comparatively preserved FLG and loricrin expression despite demonstrable barrier impairment [15]. These distinctions carry practical weight for biomarker interpretation, since a marker validated predominantly in one population may not perform equivalently across others [15].

### 3.4. Barrier Biomarker

The stratum corneum (SC), the outermost layer of the epidermis, is a central site of barrier dysfunction in AD. The SC is composed mainly of three lipid classes: ceramides (CER), cholesterol (CHOL), and free fatty acids (FFAs), along with smaller amounts of free sphingoid bases (SBs), cholesterol sulfate, and cholesterol esters [42]. Among its key barrier determinants, SC pH and SC ceramides have attracted particular interest. Elevated SC pH increases serine protease activity, interferes with normal lipid metabolism, and fosters microbiome imbalance, thereby worsening barrier impairment and inflammation [43]. Elevated SC pH reduces the activity of the acid-dependent lipid-processing enzymes β-glucocerebrosidase and acidic sphingomyelinase, which have optimal activity under acidic conditions. β-glucocerebrosidase hydrolyzes glucosylceramides to generate ceramides, while acidic sphingomyelinase hydrolyzes sphingomyelin to produce ceramides [44]. These ceramides are necessary for the organization and formation of extracellular lipid lamellae that maintain the epidermal permeability barrier [44]. As SC pH increases, reduced activity and degradation of these enzymes decrease ceramide generation, leading to the disruption of the lamellar membrane organization, contributing to increased epidermal permeability and impaired barrier function [44]. In parallel, quantitative and qualitative changes in SC ceramides further weaken barrier integrity. Because ceramides are major components of the extracellular lipid matrix responsible for limiting water loss, changes in their quantity and composition can contribute to an increase in permeability and impaired barrier function in AD [44]. Growing evidence supports SC pH and ceramides as both biomarkers of disease progression and targets for barrier-restoring therapies. Elevated SC pH is now considered closely linked to AD pathogenesis and may also contribute to the atopic march, which is known as the progression of severity of allergic diseases beginning in children. Experimental data show that combining SC acidification with PAR2 inhibition can significantly ease hapten-induced AD-like dermatitis, suggesting that increased SC pH is involved not only in disease onset and progression but also in downstream atopic comorbidities [3]. Because SC pH rises whenever the permeability barrier is compromised, regardless of the cause, gentle skin care and early control of inflammation, aimed at preventing further SC pH elevation, are critical to reducing the risk of AD development, flares, and progression along the atopic march [43]. Another barrier biomarker, transepidermal water loss (TEWL), reflects passive water movement across the stratum corneum and rises when the skin barrier is impaired. It is widely used in dermatologic research as a standard measure of barrier function and has also been investigated as a biomarker for predicting AD onset in early infancy. However, findings on the predictive value of TEWL in this context have been inconsistent [42]. TEWL may be more informative when interpreted alongside additional measures of barrier function, including SC pH, ceramide composition, and FLG-related abnormalities, rather than as an isolated biomarker. These biomarkers demonstrate significant potential for detecting skin barrier alterations in AD and show promise for identifying disease endotypes, monitoring therapeutic responses, predicting disease onset, and supporting diagnostic decision-making (Table 1).

## 4. Current Treatments

AD treatments often focus on reducing inflammation, restoring the skin barrier, and controlling pruritus. The distinct biomarker profiles that characterize different endotypes are particularly relevant for selecting molecularly targeted therapies and optimizing individualized treatment, as these endotypes may exhibit differential responses to specific interventions [37]. Current therapies can accordingly be arranged along a spectrum of mechanistic specificity: topical corticosteroids and calcineurin inhibitors act broadly across the type 2 and barrier endotypes by suppressing T-cell activation and downstream cytokine release without discriminating among them, whereas biologics and small-molecule inhibitors target defined nodes—IL-4Rα, IL-13, IL-31, and JAK-STAT signaling—identified through the biomarker profiles described above. This progression from broad immunosuppression to endotype-directed blockade frames the therapies discussed below.

### 4.1. Topical Corticosteroids

Topical corticosteroids are currently the first-line therapy for acute flare-ups, effectively reducing inflammation and itching by suppressing immune activity. They may also serve as a cost-effective and efficient treatment, with potent topical corticosteroids like fluocinolone acetonide having proven to be highly effective in enhancing clinical signs compared to controls in a meta-analysis study [45]. When applied to the skin, corticosteroids diffuse through cell membranes, bind to intracellular glucocorticoid receptors, and modulate gene transcription, downregulating proinflammatory cytokines and upregulating anti-inflammatory mediators such as lipocortin-1 [46]. This cascade suppresses prostaglandin and leukotriene synthesis by blocking arachidonic acid pathways, stabilizes mast cell and endothelial membranes, decreases histamine release, and limits T-cell migration, thereby suppressing the immune overactivity characteristic of AD [47]. Together, these actions produce rapid reductions in inflammation, pruritus, and erythema while facilitating restoration of the skin barrier. Topical corticosteroids are beneficial; however, their misuse is common. In a study by Jeziorkowska et al., patient adherence to topical corticosteroid guidelines in AD was found to be significantly lacking, with 92% of patients having modified their treatment without physician direction, leading to risks of skin atrophy, barrier dysfunction, steroid-induced acne, and increased systemic absorption [47]. Prolonged, inappropriate use of potent topical corticosteroids—most often on facial and genital skin—has also been associated with topical corticosteroid withdrawal, in which burning and stinging (the most frequently reported symptom, 65.5%) and confluent erythema (the most common sign, 92.3%) emerge upon discontinuation [48,49]. Two systematic reviews recognize it as a distinct adverse effect of TCS misuse, though the underlying evidence is of very low quality and formal diagnostic criteria have only recently been proposed [48]. Separately, children absorb proportionally more drug owing to a higher body-surface-area-to-weight ratio, raising concern for hypothalamic–pituitary–adrenal axis suppression and growth retardation; in practice, however, a systematic review found no association between mild-to-moderate potency TCS and growth abnormalities; because severe eczema independently impairs growth, any observed impairment is difficult to attribute to treatment [50].

### 4.2. Topical Calcineurin Inhibitors (TCIs)

Topical calcineurin inhibitors (TCIs) such as tacrolimus and pimecrolimus serve as steroid-sparing agents, beneficial for sensitive areas such as the face. However, these medications carry an FDA black box warning due to the potential risk of increased cancer, necessitating long-term safety monitoring [51]. TCIs inhibit calcineurin, thereby blocking T-cell activation and cytokine release in the skin. Because they do not act like steroids, they avoid some of the classic steroid side effects. They are particularly beneficial in sensitive areas, such as the face, eyelids, and other regions where steroid use is often a concern and contraindicated in specific patient populations [52]. Some side effects include a burning sensation that may appear within the first few days of use; however, this typically resolves as the skin barrier heals.

### 4.3. Phototherapy

Phototherapy is typically reserved as a second-line treatment for moderate-to-severe AD when topical therapies prove inadequate. NB-UVB and UVA1 induce apoptosis of activated T cells in the skin, thereby decreasing the number of Langerhans cells and other antigen-presenting cells [45]. Phototherapy shifts the cytokine environment by suppressing proinflammatory cytokines and increasing IL-10, thereby dampening Th2/Th22-driven inflammation in AD. Narrowband UVB is preferred in AD due to its good efficacy, tolerability, and favorable safety profile. It suppresses the Th2/Th22/Th1 pathways, normalizes epidermal hyperplasia, and improves barrier protein expression [45]. Phototherapy faces logistical and economic hurdles, including frequent required in-clinic sessions and potentially high costs when not covered by insurance. Along with this, phototherapy is contraindicated in patients with prior skin cancer diagnoses, strong family history of melanoma, or patients with photosensitivity disorders.

### 4.4. Biologics and Targeted Immunomodulatory Therapies

Advances in understanding type 2 immune dysregulation in AD has driven the development of biologic and targeted immunomodulatory therapies. These agents selectively disrupt key components of the IL-4, IL-13, IL-31, and JAK–STAT pathways, thereby reducing inflammation, pruritus, and barrier dysfunction. The approval of multiple biologics and JAK inhibitors for moderate-to-severe AD reflects a clear shift toward precision-based treatments [53]. Dupilumab, the first approved biologic for AD, is a fully human monoclonal antibody that binds IL-4 receptor alpha, thereby blocking downstream IL-4 and IL-13 signaling, two key drivers of type 2 inflammation in atopic dermatitis [53]. Clinical trials have demonstrated that dupilumab significantly improves disease severity scores such as SCORAD and EASI, and its use alongside topical therapies further enhances treatment responses. Dupilumab is particularly beneficial for patients with AD who also have comorbid conditions such as asthma or chronic sinonasal disease. Another biologic, lebrikizumab, is a monoclonal antibody targeting IL-13 that blocks its interaction with IL-13Rα1 and IL-4Rα, thereby blocking downstream IL-13 signaling. In randomized controlled trials in patients with moderate-to-severe symptoms, lebrikizumab produced significant reductions in EASI scores compared with placebo [53]. Reported adverse events were generally mild to moderate and included nasopharyngitis, headache, injection-site reactions, upper respiratory tract infections, and conjunctivitis. Phase II and III studies are ongoing to further evaluate lebrikizumab for the treatment of AD. Another option for moderate to severe AD is tralokinumab. This monoclonal antibody inhibits IL-13 by blocking IL-13Ra1 and IL-13Ra2 [54]. Tralokinumab received FDA approval in 2021 for AD, and the approval of the drug was supported by three randomized, double-blind, placebo-controlled Phase III trials that demonstrated consistent response rates higher than placebo across all trials. The most common adverse reactions included injection-site reactions, conjunctivitis, and upper respiratory tract infections [54]. Another biologic, nemolizumab, in combination with topical corticosteroids or TCIs, demonstrated efficacy, producing statistically and clinically significant reductions in inflammation and itch in adults and adolescents with moderate-to-severe AD [55]. If approved, nemolizumab could represent a valuable addition to the current therapeutic options. It works by targeting IL-31RA, thereby blocking IL-31 signaling involved in cutaneous inflammation and pruritus. By interfering with this neuroimmune itch pathway, a key driver of AD, nemolizumab helps reduce itch intensity. In the Phase III ARCADIA 1 and ARCADIA 2 trials, patients with AD treated with nemolizumab plus topical corticosteroids showed greater improvements in symptoms, pruritus, and sleep disturbance compared with those receiving placebo plus corticosteroids [46]. For patients with moderate to severe active AD who do not respond adequately to, or cannot tolerate, first-line methotrexate or other biologics, upadacitinib is an FDA-approved second-line treatment option. It works by inhibiting JAK-STAT signaling that leads to a reduction in downstream inflammatory cytokine signaling seen in AD [55]. Upadacitinib, however, should not be used in combination with other JAK inhibitors, biologic DMARDSc (infliximab, adalimumab, etanercept, abatacept, tofacitinib) or other immunosuppressants [56]. Before beginning this therapy, patients should be monitored with a tuberculosis screening, complete blood counts, liver function tests, and a lipid panel at around 12 weeks after initiation. There are several adverse effects associated with the drug; upper respiratory tract infection, elevated liver enzymes, fever and cough [56]. More recently, in September 2021, the FDA approved topical ruxolitinib cream for the treatment of mild-to-moderate AD, marking the first JAK inhibitor cream authorized for use in the United States [57]. Ruxolitinib is a selective JAK1 and JAK2 inhibitor approved in topical form for the treatment of mild-to-moderate atopic dermatitis. By competitively binding to the ATP site of JAK1 and JAK2, it disrupts JAK–STAT signaling and thereby reduces proinflammatory cytokine activity [57]. Because JAK2 also mediates erythropoietin and thrombopoietin signaling, systemic exposure can be associated with hematologic adverse effects such as anemia, neutropenia, and thrombocytopenia, although these risks are lower with topical administration compared with oral formulations [58].

## 5. OMM as a Hypothesis-Generating Neuroimmune Intervention

Osteopathic manipulative medicine (OMM) embodies the core philosophy of osteopathic medicine, viewing the body as a unit of body, mind, and spirit. The body possesses self-regulatory and self-healing mechanisms that, often with the help of OMM, can re-establish balance across interconnected body systems to maintain health. Through hands-on techniques, OMM strives to correct somatic dysfunctions, improve motion, enhance circulation, and support the body’s innate healing capacity. Rather than targeting symptoms alone, OMM aims to address the whole person, attempting to restore homeostasis among the neurological, circulatory, lymphatic, and musculoskeletal systems to improve overall health.

One proposed mechanism involves the brain–skin–autonomic axis, through which psychological stress and an increase in sympathetic activity may possibly influence the neuroimmune signaling pathway and exacerbate pruritis. OMM has been explored for potential effects on the autonomic activity in non-AD populations, providing a basis to examine whether similar physiologic changes can be relevant to AD. A possible hypothesis is that a variety of OMM actions may reduce sympathetic tone and influence the stress-related cortisol and catecholamine signaling. These changes could, in turn, influence neurogenic itch and stress-induced exacerbations, leading to a possible reduction in future barrier injury. This pathway may provide an indirect mechanism through which autonomic regulation could interact with processes that are deemed relevant to AD, rather than suggesting a direct effect of OMM on cutaneous inflammatory pathways. However, these relationships have not been directly demonstrated in patients with AD, and the proposed pathway remains a testable hypothesis requiring mechanistic and clinical investigation.

**Figure 1:**
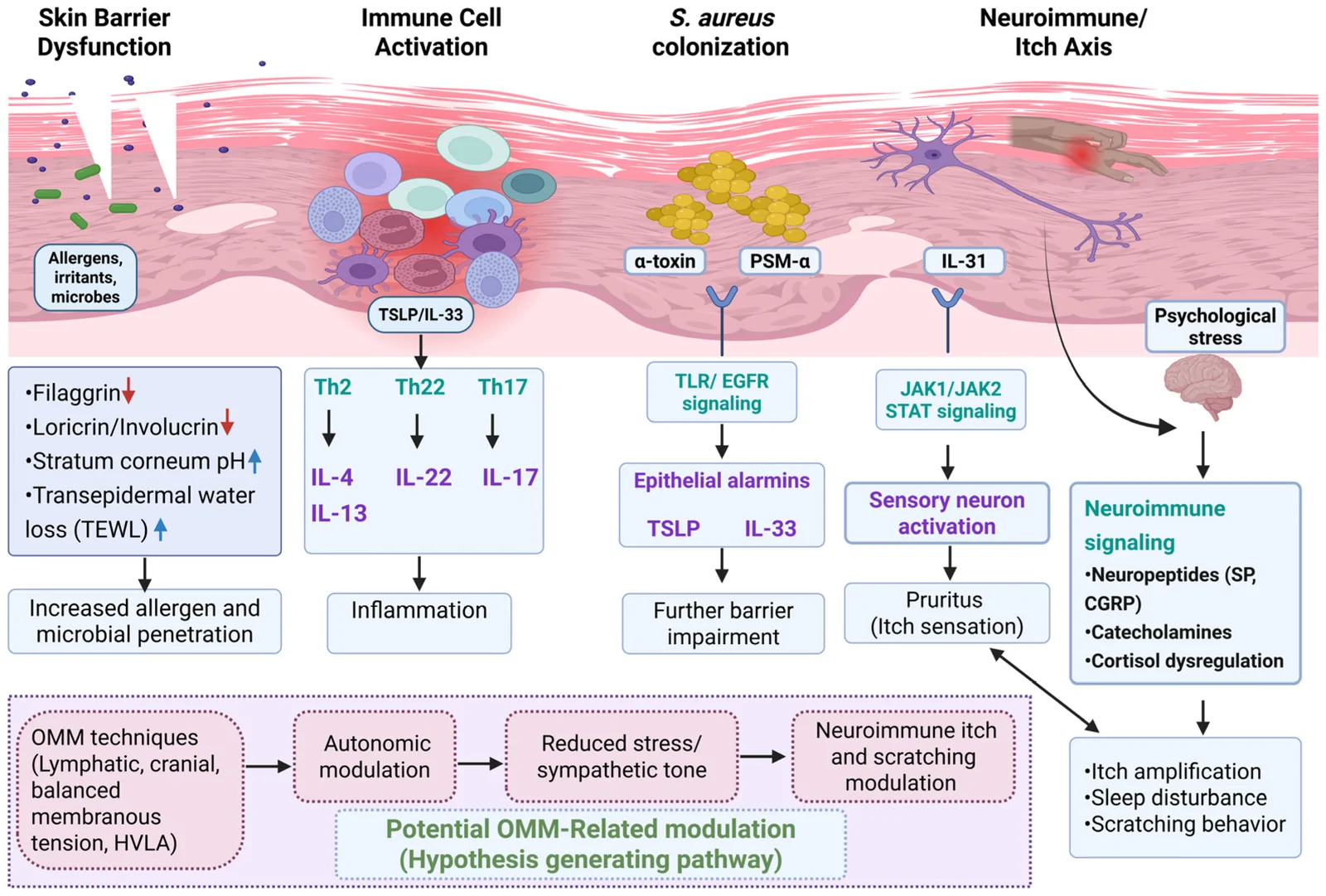
Integrated molecular, microbial, and neuroimmune mechanisms underlying atopic dermatitis. Disruption of the epidermal barrier, including alterations in filaggrin, structural proteins, stratum corneum pH, ceramides, and transepidermal water loss, promotes allergen and microbial penetration. *Staphylococcus aureus* colonization and microbial virulence factors can contribute to keratinocyte activation and induction of epithelial alarmins, including thymic stromal lymphopoietin (TSLP) and IL-33. These signals promote type 2 and other inflammatory pathways, including Th2-, Th22-, and Th17-associated responses, which further impair barrier function. Neuroimmune interactions, including IL-31/IL-31 receptor signaling, contribute to sensory neuron activation and chronic pruritus. Scratching further disrupts the epidermal barrier, creating a self-perpetuating itch–inflammation cycle. Stress-related autonomic signaling may additionally amplify neuroimmune itch. The potential effects of osteopathic manipulative medicine (OMM) on this axis are shown as a hypothesis-generating pathway and require direct mechanistic and clinical validation in patients with AD. Solid line: established mechanisms; dotted line: hypothesized/requires validation in AD. (Created using BioRender). (Red down arrow represent decrease, blue upward arrow represent increase, black arrow represent chain of events/sequence of events).

Lymphatic osteopathic manipulative treatment (OMT) is a hands-on, physician-delivered intervention in which osteopathic physicians use manual techniques—such as thoracic inlet release, rib raising, thoracic pump, and pedal pump—to address somatic dysfunction of the musculoskeletal system and related structures with the goal of influencing lymphatic circulation (Figure 1) [59,60]. These techniques have been shown to influence lymphatic drainage by removing mechanical barriers, mobilizing central lymphatic flow, and increasing peripheral return [61]. Although lymphatic OMT has been reported to influence lymphatic flow in non-AD experimental settings [62], there is currently no direct evidence that these techniques alter cutaneous cytokine concentrations or molecular endotypes in patients with AD. Therefore, any potential effects of lymphatic OMT on inflammatory processes in AD should be considered hypothesis-generating and require direct investigation.

A potentially more direct avenue for investigation is the brain–skin–autonomic axis, through which modulation of stress and autonomic activity could influence neuroimmune signaling and pruritus. Autonomic balancing techniques such as rib raising, suboccipital release and sacral rocking have been investigated for their potential effects on autonomic nervous system activity (Figure 1) [63]. Rib raising, which influences the thoracic sympathetic chain, has been seen to reduce sympathetic outflow and increase heart rate variability, a validated marker of parasympathetic activity. Rib raising has been seen to decrease skin conductance and decrease heart rate, reflections of a shift towards parasympathetic autonomic balance. Suboccipital release, which alleviates tension in the upper cervical region affecting the vagus nerve, has been associated with a decrease in sympathetic tone. Along with this, sacral rocking influences parasympathetic outflow through the pelvic splanchnic nerves (S2–S4) and can increase relaxation, improve pelvic circulation and increase autonomic balance [63]. Together, these findings raise the hypothesis that the use of OMM as a stress reduction and autonomic modulator could decrease sympathetic tone and possibly influence stress-related cortisol and catecholamine signaling. These changes may reduce neurogenic itch and stress-induced exacerbations, with secondary effects on scratching and subsequent barrier injury.

In a previous randomized controlled trial, Ruffini et al. examined whether OMT measurably alters autonomic nervous system activity, as assessed by heart rate variability (HRV) [64]. OMM treatment was associated with a significant increase in parasympathetic activity. Additionally, there was a reduction in sympathetic markers when compared with sham therapy and no-intervention controls. Although these findings suggest that OMM may influence autonomic physiology, these effects were not evaluated in patients with AD. Therefore, they provide a mechanistic basis for investigating whether OMM-associated autonomic modulation could influence stress-related neuroimmune pathways in AD.

Importantly, no randomized controlled trials have evaluated OMT in AD. Therefore, the proposed role of OMM in AD should be regarded as hypothesis-generating rather than evidence of therapeutic efficacy. Further randomized controlled trials should compare OMT protocols with appropriate controls to evaluate clinical and mechanisitic outcomes. Some clinical endpoints could include EASI, SCORAD, TEWL pruritus severity, quality of life and cytokine and chemokine profiles. Additional outcomes such as hear rate variability, skin conductance, and cortisol or catecholamine levels could be considered as testable outcomes. Thus, the proposed pathway linking OMM-associated stress reduction and autonomic modulation of neuroimmune signaling, neurogenic itch, scratching and secondary barrier injury represents a testable biological hypothesis rather than an established theraputic mechanism (Figure 1).

## 6. Discussion

AD impacts over 30 million people in the United States, contributing to a significant economic burden exceeding $5 billion annually [65]. This includes both direct costs, such as prescription medications, healthcare visits, and hospitalizations, and indirect costs, including lost productivity and overall reduced quality of life. Inadequate or delayed treatment of AD is associated with increased emergency department visits and hospitalizations, highlighting the importance of early intervention and optimized disease management in reducing healthcare utilization. Recognizing the economic impact of AD is essential for delivering high-quality treatment and optimizing patient outcomes [65]. Given that stress levels can influence the severity of symptoms seen in individuals with AD, it is important to explore complementary approaches. One study demonstrated that OMM and stress management techniques have a significant impact on the treatment of atopic dermatitis [66]. Osteopathic medicine and dermatology share foundational principles that emphasize a holistic approach to treating conditions. This integration fosters improved patient relationships, the development of innovative treatment plans, enhanced stress management, and more personalized care [67].

AD is believed to arise from a complex interplay between skin barrier integrity, Th2 immune cell dysregulation, autonomic imbalance and lymphatic dysfunction. Barrier dysfunction leads to allergen access and skewed Th2 immune response and increased levels of IgE and eosinophils. Additionally, *S. aureus* overgrowth has also been associated with exacerbation of AD pathogenesis. Similarly, autonomic imbalance and impaired lymphatic drainage contribute to neurological itch and cytokine accumulation. The treatment of atopic dermatitis poses several challenges despite advances in topical, systemic, and biologic therapies. The condition has been viewed as highly heterogeneous, with numerous variations in immune endotypes, microbiome alterations, environmental factors, and even psychosocial triggers [68]. This heterogeneity contributes to the inconsistent treatment responses often seen in patients with AD, leading to many obstacles for long-term remission and treatment. Although biomarker-driven therapies have improved outcomes for many patients, persistent pruritus and recurrent flares highlight the need for additional supportive treatment approaches. Current treatment approaches focus on suppressing inflammation or blocking specific cytokine signaling pathways. While these strategies can be effective, they may not fully address the root cause of the pathogenesis of atopic dermatitis, including autonomic imbalance, impaired lymphatic clearance, and stress-induced neuroimmune dysregulation. OMM offers a patient-centered approach that aligns with the multifactorial nature of atopic dermatitis. OMM focuses on the relationship between structure and function to optimize autonomic balance, lymphatic circulation, and overall physiological regulation, rather than targeting isolated molecular pathways [68]. OMT may help reduce upstream drivers of inflammation and improve the physiological conditions required for inflammatory resolution. By using osteopathic manipulative techniques to optimize autonomic balance and lymphatic clearance, OMM may increase responsiveness to existing conventional therapies and aid long-term resolution of atopic dermatitis by sustained improvements in disease severity (e.g., SCORAD/EASI), pruritus, sleep quality, and overall quality of life.

## 7. Conclusions and Future Directions

AD is a complex, heterogeneous disease driven by immune dysregulation, barrier dysfunction, genetic factors, as well as environmental and psychosocial influences, making a single curative strategy improbable. Advances in biomarker discovery have begun to refine diagnosis, stratify endotypes, and guide targeted therapies, improving outcomes for many patients but not fully addressing persistent symptoms such as pruritus, recurrent flares, and long-term disease control. Within this context, osteopathic manipulative medicine offers a biologically plausible adjunct to conventional care by targeting autonomic balance, lymphatic circulation, and overall physiological regulation—mechanisms that directly interface with systemic inflammation and skin barrier restoration. Although current evidence for OMT in atopic dermatitis is limited, its integration into individualized, biomarker-informed treatment strategies may help reduce upstream drivers of inflammation and enhance responsiveness to existing therapies, as evidenced by emerging biomarker data and targeted therapies that underscore the need for integrative, endotype-aware care. Within this framework, osteopathic manipulative medicine represents a biologically plausible, hypothesis-driven adjunct that targets autonomic balance, lymphatic circulation, and systemic inflammatory tone and can be explicitly tested in clinical settings. To move beyond conceptual plausibility, future studies must prioritize rigorous mechanistic and clinical trials that evaluate OMM as part of individualized, biomarker-informed treatment algorithms. Future studies could incorporate clinical outcomes including EASI, SCORAD, pruritus NRS, sleep quality, and DLQI alongside specific molecular and physiologic endpoints. Barrier function could be assessed using transepidermal water loss (TEWL), stratum corneum pH, ceramide profiles, and expression of barrier proteins such as filaggrin, loricrin, and involucrin. Immune and inflammatory endpoints could include IL-4, IL-13, IL-31, TSLP, IL-33, CCL17/TARC, and periostin. Neuroendocrine and autonomic measures, including heart rate variability (HRV), cortisol, catecholamines, and sympathetic skin response, could be evaluated to test the possible relationship between autonomic regulation and neuroimmune activity. Correlating these molecular, barrier, and autonomic measures with clinical outcomes could allow for future studies to determine whether OMM may be associated with measurable changes in AD-related biological pathways and test the proposed mechanistic framework. Such studies could clarify how OMM may complement conventional therapies, enhance treatment responsiveness, and better align AD management with the principles of precision and whole-person medicine.

## Figures and Tables

**Table 1:** Major AD endotypes, their representative biomarkers, biological implications, and potential therapeutic targets.

AD Endotype	Representative Biomarkers	Biological Implication	Potential Therapeutic Relevance
Type 2	IL-4, IL-13, CCL17/TARC, periostin	Barrier dysfunction/inflammation	Dupilumab, IL-13 blockade
Itch/neuroimmune	IL-31, IL-31RA	Pruritus	Nemolizumab
Barrier	FLG, ceramides, TEWL, SC pH	Barrier dysfunction	Barrier restoration
Th17/Th22	IL-17, IL-22	Chronic inflammation/epidermal hyperplasia	Endotype-specific targeting
Microbial	*S. aureus*, microbial diversity, toxins	Dysbiosis/alarmin activation	Antimicrobial/barrier approaches
Systemic/autonomic	Cortisol, catecholamines, HRV	Stress/neuroimmune component	OMM: investigational hypothesis

## Data Availability

No new data were created or analyzed in this study. Data sharing is not applicable to this article.

## Abbreviations

The following abbreviations are used in this manuscript:

AD	atopic dermatitis
AMPs	antimicrobial peptides
ASC	apoptosis-associated speck-like protein containing a CARD
BNP	brain natriuretic peptide
CCL17	C-C motif chemokine ligand 17 (TARC)
CCL18	C-C motif chemokine ligand 18 (PARC)
CCL22	C-C motif chemokine ligand 22 (MDC)
CCL26	C-C motif chemokine ligand 26 (eotaxin-3)
CCL27	C-C motif chemokine ligand 27 (CTACK)
CER	ceramide
CGRP	calcitonin gene-related peptide
CHOL	cholesterol
COX-2	cyclooxygenase-2
CTACK	cutaneous T-cell-attracting chemokine (CCL27)
DLQI	Dermatology Life Quality Index
DMARDs	disease-modifying antirheumatic drugs
DPP-4	dipeptidyl peptidase-4
DNFB	2,4-dinitrofluorobenzene
DSF70	dense fine speckled 70 autoantigen
EASI	Eczema Area and Severity Index
EGFR	epidermal growth factor receptor
FDA	U.S. Food and Drug Administration
FFA	free fatty acid
FLG	filaggrin
HPA	hypothalamic–pituitary–adrenal axis
HRV	heart rate variability
IFN-γ	interferon gamma
IgE	immunoglobulin E
IL	interleukin (IL-1β, IL-4, IL-5, IL-10, IL-13, IL-17, IL-22, IL-25, IL-31, IL-33)
IL-1R	interleukin-1 receptor
IL-4Rα	interleukin-4 receptor alpha
IL-13Rα1	interleukin-13 receptor alpha 1
IL-13Rα2	interleukin-13 receptor alpha 2
IL-31RA	interleukin-31 receptor A
IL-36R	interleukin-36 receptor
ILC2	group 2 innate lymphoid cell
JAK	Janus kinase (JAK1, JAK2)
LDH	lactate dehydrogenase
LEKTI	lymphoepithelial Kazal-type-related inhibitor
MCP-3	monocyte chemoattractant protein-3
MDC	macrophage-derived chemokine (CCL22)
MyD88	myeloid differentiation primary response 88
NB-UVB	narrowband ultraviolet B
NF-κB	nuclear factor kappa B
NOS2	inducible nitric oxide synthase
NP3	NP3 sensory neuron
NRS	numeric rating scale
OMM	osteopathic manipulative medicine
OMT	osteopathic manipulative treatment
OSMR	oncostatin M receptor
PAR1	protease-activated receptor 1
PAR2	protease-activated receptor 2
PARC	pulmonary and activation-regulated chemokine (CCL18)
PSMα	phenol-soluble modulin alpha
SB	sphingoid base
SC	stratum corneum
SCORAD	SCORing Atopic Dermatitis
SPINK5	serine peptidase inhibitor Kazal type 5
STAT	signal transducer and activator of transcription (STAT1, STAT3, STAT5)
TARC	thymus and activation-regulated chemokine (CCL17)
TCI	topical calcineurin inhibitor
TCM	traditional Chinese medicine
TEWL	transepidermal water loss
Th	T-helper cell (Th1, Th2, Th17, Th22)
TLRs	Toll-like receptors (TLR2, TLR8, TLR9)
TNF-α	tumor necrosis factor alpha
TNF-β	tumor necrosis factor beta
TRPV1	transient receptor potential vanilloid 1
TSLP	thymic stromal lymphopoietin
UVA1	ultraviolet A1
VEGF	vascular endothelial growth factor
VEGFR-3	vascular endothelial growth factor receptor 3
VIP	vasoactive intestinal peptide
